# Monitoring Changes in Oxygen Muscle during Exercise with High-Flow Nasal Cannula Using Wearable NIRS Biosensors

**DOI:** 10.3390/bios13110985

**Published:** 2023-11-13

**Authors:** Felipe Contreras-Briceño, Maximiliano Espinosa-Ramírez, Augusta Rivera-Greene, Camila Guerra-Venegas, Antonia Lungenstrass-Poulsen, Victoria Villagra-Reyes, Raúl Caulier-Cisterna, Oscar F. Araneda, Ginés Viscor

**Affiliations:** 1 Laboratory of Exercise Physiology, Department of Health Science, Faculty of Medicine, Pontificia Universidad Católica de Chile, Av. Vicuña Mackenna #4860, Santiago 7820436, Chile; maespinosa@uc.cl (M.E.-R.); augusta.rivera@uc.cl (A.R.-G.); camila.guerra@uc.cl (C.G.-V.); antonia.lungenstrass@uc.cl (A.L.-P.); vavillagra@uc.cl (V.V.-R.); 2 Millennium Institute for Intelligent Healthcare Engineering (iHEALTH), Av. Vicuña Mackenna #4860, Santiago 7820436, Chile; 3 Department of Informatics and Computing, Faculty of Engineering, Universidad Tecnológica Metropolitana, Av. José Pedro Alessandri #1242, Santiago 7800002, Chile; rcaulier@utem.cl; 4 Laboratory of Integrative Physiology of Biomechanics and Physiology of Effort, Kinesiology School, Faculty of Medicine, Universidad de los Andes, Av. Monseñor Álvaro del Portillo 12455, Santiago 7620001, Chile; ofaraneda@miuandes.cl; 5 Secció de Fisiologia, Departament de Biologia Cel·lular, Fisiologia i Immunologia, Facultat de Biologia, Universitat de Barcelona, Av. Diagonal #643, 08028 Barcelona, Spain; gviscor@ub.edu

**Keywords:** near-infrared spectroscopy, exercise, rehabilitation, wearable, optical

## Abstract

Exercise increases the cost of breathing (COB) due to increased lung ventilation ($\dot{V}$E), inducing respiratory muscles deoxygenation (∇SmO_2_), while the increase in workload implies ∇SmO_2_ in locomotor muscles. This phenomenon has been proposed as a leading cause of exercise intolerance, especially in clinical contexts. The use of high-flow nasal cannula (HFNC) during exercise routines in rehabilitation programs has gained significant interest because it is proposed as a therapeutic intervention for reducing symptoms associated with exercise intolerance, such as fatigue and dyspnea, assuming that HFNC could reduce exercise-induced ∇SmO_2_. SmO_2_ can be detected using optical wearable devices provided by near-infrared spectroscopy (NIRS) technology, which measures the changes in the amount of oxygen bound to chromophores (e.g., hemoglobin, myoglobin, cytochrome oxidase) at the target tissue level. We tested in a study with a cross-over design whether the muscular desaturation of *m.vastus lateralis* and *m.intercostales* during a high-intensity constant-load exercise can be reduced when it was supported with HFNC in non-physically active adults. Eighteen participants (nine women; age: 22 ± 2 years, weight: 65.1 ± 11.2 kg, height: 173.0 ± 5.8 cm, BMI: 21.6 ± 2.8 kg·m^−2^) were evaluated in a cycle ergometer (15 min, 70% maximum watts achieved in ergospirometry ($\dot{V}$O_2_-peak)) breathing spontaneously (control, CTRL) or with HFNC support (HFNC; 50 L·min^−1^, fiO_2_: 21%, 30 °C), separated by seven days in randomized order. Two-way ANOVA tests analyzed the ∇SmO_2_ (*m.intercostales* and *m.vastus lateralis)*, and changes in $\dot{V}$E and ∇SmO_2_·$\dot{V}$E^−1^. Dyspnea, leg fatigue, and effort level (RPE) were compared between trials by the Wilcoxon matched-paired signed rank test. We found that the interaction of factors (trial × exercise-time) was significant in ∇SmO_2_-*m.intercostales*, $\dot{V}$E, and (∇SmO_2_-*m.intercostales*)/$\dot{V}$E (*p* < 0.05, all) but not in ∇SmO_2_-*m.vastus lateralis*. ∇SmO_2_-*m.intercostales* was more pronounced in CTRL during exercise since 5′ (*p* < 0.05). Hyperventilation was higher in CTRL since 10′ (*p* < 0.05). The ∇SmO_2_·$\dot{V}$E^−1^ decreased during exercise, being lowest in CTRL since 5′. Lower dyspnea was reported in HFNC, with no differences in leg fatigue and RPE. We concluded that wearable optical biosensors documented the beneficial effect of HFNC in COB due to lower respiratory ∇SmO_2_ induced by exercise. We suggest incorporating NIRS devices in rehabilitation programs to monitor physiological changes that can support the clinical impact of the therapeutic intervention implemented.

## 1. Introduction

It is estimated that energy cost associated with spontaneous breathing at rest primarily due to contraction of respiratory muscles (RMs) (cost of breathing, COB) varies from 2–5% in healthy subjects [1,2] to 10–15% in patients with chronic cardiorespiratory dysfunctions (CCDs) [3,4]. 

The exercise-induced increase of minute ventilation ($\dot{V}$E) implies an increase of COB (to 25–45%) with consequently increased blood flow ($\dot{Q}$), nutrients, and oxygen (O_2_) to RMs [1,5]. This redistribution of $\dot{Q}$ can limit O_2_ supply to locomotor muscles and, thus, be recognized as a respiratory limitation to exercise in athletes [6,7], non-active subjects [8,9], and patients with CCDs (e.g., COPD, arterial hypertension, asthma) [10,11,12]. 

A COB increase has been associated with poor effort tolerance due to dyspnea and/or muscle leg fatigue [2,13]. To reduce COB and, thus, improve the exercise time involved in rehabilitation programs and achieve beneficial clinical outcomes, the use of non-invasive ventilatory support systems (e.g., non-invasive ventilation (NIV) and high-flow nasal cannula (HFNC)) during physical training has been employed by medical professionals [14,15,16,17,18,19]. 

In this context, previous studies have reported that patients with COPD who exercised with HFNC support exhibited an extended exercise time and greater peripheral oxygen extraction than those using traditional oxygen equipment [16,20], suggesting a potential decrease in COB associated with HFNC support. Conversely, a recent meta-analysis of this therapeutic tool in respiratory rehabilitation of patients with CCDs did not show a positive impact on lung mechanics, breathing patterns, and exercise capacity [21]. The low-to-moderate intensity during the physical training of these studies could be why positive effects were unhighlighted. 

Elucidating whether the airflow given by HFNC in exercise intervention can impact lower COB, and thus, increase exercise tolerance is crucial to improve the effectiveness of this novel therapeutic intervention in rehabilitation programs. However, the gold-standard method to register work of breathing (WOB) is based on invasive procedures, such as inserting an esophageal balloon catheter [21], making it difficult to use routinely. 

A novel method employed to estimate the COB is by the non-invasively recording of changes in muscular oxygen saturation levels (SmO_2_) in RMs, specifically at *m.intercostales* (SmO_2_-*m.intercostales*) during actions that require more RMs force production (e.g., exercise, respiratory muscle training) [22,23,24,25,26]. 

The SmO_2_ record is based on near-infrared spectroscopy (NIRS) technology and was described for the first time in 1977 by Jöbsis for monitoring cerebral oxygenation [27]. NIRS is based on the modified Beer–Lambert’s law (Equation (1)), which considers the dispersion of the nature of the tissues and their geometry.

(1) $$A=\log\;\frac{I}{I0}\;=\varepsilon \;\left[C\right]L*DPF+G$$

Equation (1). Modified Beer–Lambert’s law, where “A” is the absorption, “I” is the luminous intensity, “ε” is the extinction coefficient for the light absorbing compound of interest, $“\left[C\right]”$ is the concentration of the compound of interest, “L” is the distance between the source and detector diodes, “DPF” the differential path length factor, and “G” is the factor reflecting non-absorption. 

Some commercial optical wearable devices can easily detect the amount of oxygen bound to chromophores (i.e., hemoglobin (Hb), myoglobin (Mb), cytochrome oxidase (cyt_ox_)) at the target tissue level [28,29,30]. The concentration of cyt_ox_ in mammalian muscle is likely ~5% or less compared with Hb and Mb, suggesting the primary sources of NIRS signals are from changes in Hb and Mb [28]. The contribution of Mb to the signal varies from 20% to 30% at rest and 50% during repeated contractions [31]. Thus, it is expected that in clinical exercise physiology investigations, the muscle measurements obtained with NIRS technology allude to changes in Hb as the primary signal source [28]. 

In studies with exercise as part of the experimental protocol, optical wearable biosensors with NIRS technology have been positioned mainly in locomotor muscles (e.g., *m.vastus lateralis*, *m.tibialis anterior*, and *m.gastrocnemius*) [32,33] to identify effort intensity. Our research group has examined the changes in SmO_2_ in RMs (*m.intercostales*) and *m.vastus lateralis* during an incremental exercise in athletes [22,34,35], untrained subjects [36], and patients with Fontan circulation [37], showing a consistent inverse association between a drop in SmO_2_-*m.intercostales* (∇SmO_2_-*m.intercostales*) and increased ventilatory variables, aspects more notorious at moderate-to-high exercise intensities, where to compensate for the metabolic acidosis, the respiratory system demand boosts exponentially. 

The SmO_2_ is based on changes in the oxyhemoglobin ([*O_2_Hb*]) and deoxyhemoglobin ([*HHb*]) (Equation (2)).

(2) $$SmO_{2}\;(\%)=\frac{O_{2}Hb}{O_{2}Hb+HHb}\times 100$$

Equation (2). Muscle oxygen saturation level (*SmO_2_*) expressed as percent (%) calculating oxyhemoglobin (*O_2_Hb*) and deoxyhemoglobin (*HHb*) measured at the microvascular level. 

To our knowledge, only three studies have evaluated changes in SmO_2_ at RMs and locomotor muscles during exercise supported with non-invasive airflow (NIV and HFNC). First, Fang et al. [20] reported lower exercise-induced deoxygenated hemoglobin in *m.vastus lateralis* in patients with COPD supported with HFNC (flow: 30 to 45 L·min^−1^; additional O_2_ to maintain an SpO_2_ > 92%). Likewise, da Luz et al. [38,39] reported that NIV during constant work-rate exercise led to the unloading of RMs, inducing a greater O_2_ supply to locomotor muscles, reducing fatigue, and sustaining longer exercise time both in healthy subjects and patients with CCDs. However, if high-intensity exercise supported with HFNC decreases exercise-induced COB, defined as a reduced ∇SmO_2_-*m.intercostales*, it has not been fully explored.

This study aimed to evaluate the effect of constant exercise at high intensity supported with HFNC on COB, assessed as the oxygen saturation levels of accessory RMs. We hypothesized that the changes in SmO_2_-*m.intercostales* during exercise supported with HFNC are less pronounced than when breathing spontaneously, an aspect that may be associated with less exercise hyperventilation and, consequently, fewer symptoms of exercise intolerance. Elucidating the above would allow supporting the use of HFNC in rehabilitation programs to develop exercise intensities associated with better performance with no COB that entails respiratory limitation and arrest of the progression of exercise.

## 2. Materials and Methods

### 2.1. Study Design and Participants

Eighteen healthy adults (from 20 to 24 years) completed all the measurements declared in this analytic study with a cross-over design. Participants were recruited from the community through advertisements and email invitations. The inclusion criterion was for subjects to be non-physically active to reduce a bias attributed to well-trained RMs and good exercise performance (less than three times per week of physical activity at moderate-to-high intensity (65% or more of the maximal theoretical value of heart rate according to the formula: 220−years)), and exclusion criteria were the following: any dysfunction of cardiovascular, respiratory, musculoskeletal systems; consumption of legal or illegal drugs (e.g., tobacco, alcohol); use of chronic medication (e.g., corticosteroids); impossibility to execute the exercise protocol; and an acute infectious or inflammation process in the two weeks prior to tests. To assess the effect of HFNC on physiological responses, the completion of a constant-load work test at high intensity, as the literature argues, is more suitable than an incremental exercise or a constant submaximal protocol [40,41]. 

The study was performed following the Declaration of Helsinki and was approved by the Institutional Ethics Committee (project nº 220608010). Participants were informed of the possible risks and benefits of participating in the study and signed the informed consent before starting the protocol.

### 2.2. Procedures

The assessments were done in the Laboratory of Exercise Physiology of the Pontificia Universidad Católica de Chile under controlled environmental conditions (20 ± 2 °C; relative humidity 40 ± 2%) and on a fixed schedule (09:00 to 12:00 h). All participants were instructed to sleep at least seven hours the night before the evaluations and not to consume any stimulant substances (e.g., alcohol, caffeine) nor perform exercise 24 h before. 

This study consisted of three sessions separated by seven days. In the first session, anthropometric (body weight, height, BMI), spirometry (Microlab, model ML3500, CareFusion^®^, San Diego, CA, USA), and exercise capacity (peak oxygen consumption, $\dot{V}$O_2_-peak) assessments were completed. In the following sessions, participants randomly performed two constant-load exercise tests (CLETs) at 70% of the maximal load achieved previously in the $\dot{V}$O_2_-peak test (CTRL = breathing spontaneously (21% fiO_2_), and HFNC = breathing with the support of HFNC (model Airvo2, Fisher & Paykel Healthcare Ltd., Auckland, New Zealand; flow at 50 L·min^−1^, air temperature at 30 °C, 21% fiO_2_) for 15 min. When participants were supported with HFNC during the exercise phase, they also completed the previous rest and warm-up phases and the following cool-down phase with the same ventilatory support setting. 

**Figure 1:**
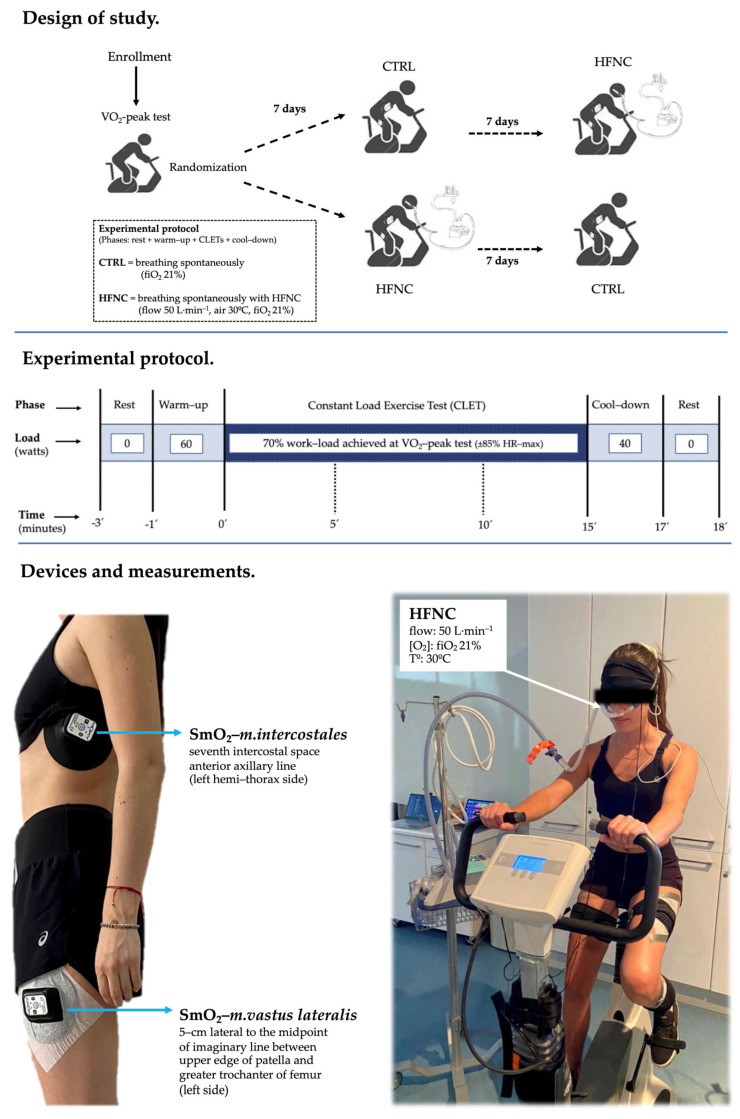
Experimental model.

During CLETs, heart rate, SpO_2_, and symptoms (dyspnea, leg fatigue, and the effort level or rate of perceived exertion (RPE) were assessed by a modified Borg’s scale) were recorded. During minute 1, minutes 3 to 5, 8 to 10, and 13 to 15 (defined as events 0′, 5′, 10′, and 15′, respectively), the exhaled gases (oxygen consumption ($\dot{V}$O_2_) and (carbon dioxide ($\dot{V}$CO_2_)) and ventilatory variables ((lung ventilation ($\dot{V}$E), respiratory rate (RR), and tidal volume (Vt)) were recorded by the ergoespirometer for registering the changes in cardio-ventilatory variables during CLETs. Figure 1 shows the study design.

### 2.3. Peak Oxygen Consumption ($\dot{V}$O_2_-Peak)

The $\dot{V}$O_2_-peak was assessed by analyzing $\dot{V}$E, RR, Vt$,\dot{V}$O_2,_ and $\dot{V}$CO_2_ by the *breath-by-breath* method (Cosmed Quark-CPET, Rome, Italy) and expressed under standard temperature pressure dry air (STPD), while the participants completed an incremental exercise until voluntary exhaustion despite verbal stimuli (respiratory quotient 1.20 ± 0.05) [42,43]. The exercise test was performed on a cycle ergometer magnetically braked (ViaSprint 150P, Ergoline GmH, Traunstein, Germany). The ergospirometer was calibrated according to the manufacturer’s instructions, using calibration gas (5.00% CO_2_, 15.99% O_2_, and the remainder nitrogen). 

The protocol consisted of a 2 min rest, 3 min warm-up period at 60 watts (W), followed by the exercise phase that started at 100 W and increased 20 W every 2 min. The participants were requested to maintain a cadence between 70 and 90 rpm during the test. The $\dot{V}$O_2_-peak was calculated as the highest value obtained during the last 30 s of the test despite increasing the exercise intensity (<150 mL·min^−1^ of exercise) [44]. A cool-down of 3 min of submaximal exercise (40 W) was performed before stopping the test.

### 2.4. Muscle Oxygen Saturation (SmO_2_)

**Figure 2:**
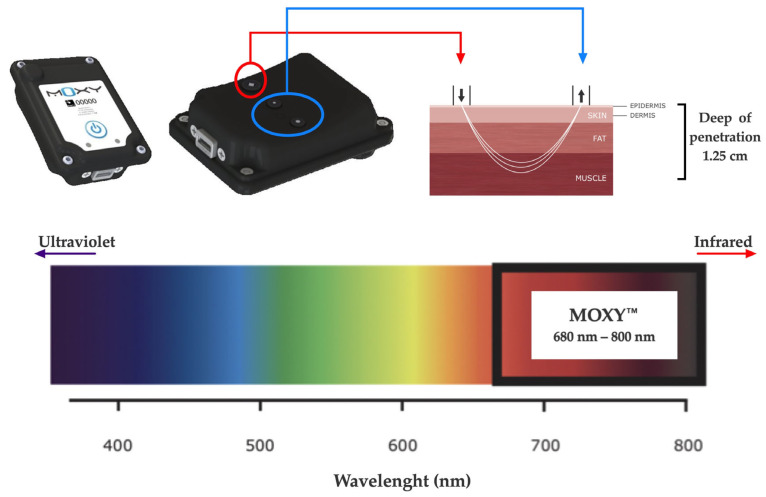
NIRS device used (MOXY^®^).

The SmO_2_ was evaluated during both CLETs by continuous-wave near-infrared spectroscopy (NIRS, 650 to 950 nm) using non-invasive devices (MOXY, Fortiori, Design LLC, Hutchinson, MN, USA) (see Figure 2). These devices use four wavelengths (680, 720, 760, and 800 nm) to assess absorbency via modified Beer–Lambert law. It measures the absorbance of near-infrared light by oxygenated hemoglobin and myoglobin (oxy (Hb + Mb)) as well as deoxygenated hemoglobin and myoglobin (deoxy [Hb + Mb]) at a microvascular level [28,32]. The light can penetrate the measured tissues with a depth of 1.25 cm as the maximum, considering the 1.25 to 2.50 cm distance between diodes (emitter and receptors) [45].

The raw SmO_2_ data were subjected to a low pass (<0.1 Hz) to remove noises associated with body movement during the exercise protocol and spontaneous slow hemodynamic oscillations. From these values, SmO_2_ was calculated using PeriPedal^®^ software (PeriPedal, Indianapolis, IN, USA) at a sampling frequency of 2 Hz from the *m.intercostales* (SmO_2_-*m.intercostales*) and *m.vastus lateralis* (SmO_2_-*m.vastus lateralis*), according to previous protocols [22,23,25,26,34,35,36,46]. 

Briefly, to record SmO_2_-*m.intercostales*, a MOXY was positioned in the seventh intercostal space of the anterior axillary line of the left hemi-thorax side, and for recording SmO_2_-*m.vastus lateralis*, a second MOXY was positioned in the left limb, 5 cm lateral to the midpoint of the line between the superior patella edge and greater trochanter of the femur. These devices were fixed to the skin with double-sided adhesive tape (avoiding covering the emitting and receiving diodes), and, at the same time, they were covered with a black case fixed with Fixomull™ to avoid displacement during exercise, according to the manufacturer’s suggestions.

### 2.5. Data Analysis 

Given the variability of SmO_2_ values when data are expressed as raw values (%), we standardized to the arbitrary unit (a.u.), considering 1.0 as the value of SmO_2_ at the start of the exercise phase (minute 0, 0′). To analyze changes in variables during CLETs, we selected the minutes 5, 10, and 15 (5′, 10′, and 15′) of the exercise phase, and these values were compared with 0′. The selected value of each time exercise (0′, 5′, 10′, and 15′) corresponds with the average of the previous 30 s. The same procedure was applied to other variables evaluated. Also, changes in the ratio SmO_2_-*m.intercostales*/$\dot{V}$E during CLETs were analyzed.

### 2.6. Statistical Analysis 

The normality of data was evaluated using the Shapiro–Wilk test. Descriptive variables are shown as mean ± standard deviation. The sample size was calculated using G*Power (version 3.1; Dusseldorf, Germany) from data extracted from pilot evaluations. Considering ∆SmO_2_-*m.intercostales* as the primary outcome, an effect size of 0.60, a power of 80%, and 95% confidence provided an estimated requirement of 14 participants. The two-way mixed ANOVA test analyzed the differences between CLETs (CTRL vs. HFNC). Dyspnea, leg fatigue, and RPE were compared between conditions by the Wilcoxon matched-paired signed rank test. Statistical significance was set at *p* < 0.05. The statistical analysis was performed using the GraphPad Prism (version 10.1.0; San Diego, CA, USA). 

## 3. Results

### 3.1. Participants’ Characteristics

All participants showed lung function and aerobic capacity according to normal values and the inclusion criterion. Also, all participants completed both trials, pedaling to target intensity. Nobody showed values of SpO_2_ lower than 95% (see Table 1).

### 3.2. Changes in Physiological Variables during Exercise

The dispersion of SmO_2_ values at rest was analyzed by the coefficient of variation for *m.intercostales* (8.95% [5.30 to 12.60] as mean and 95% CI) and *m.vastus lateralis* (5.76% [3.28 to 7.42%] as mean and 95% CI), which were less than 9% and 6%, respectively. Also, a posterior analysis was performed to identify outliers, using the iterative Grubbs method (alpha: 0.05), which did not report outliers. 

**Figure 3:**
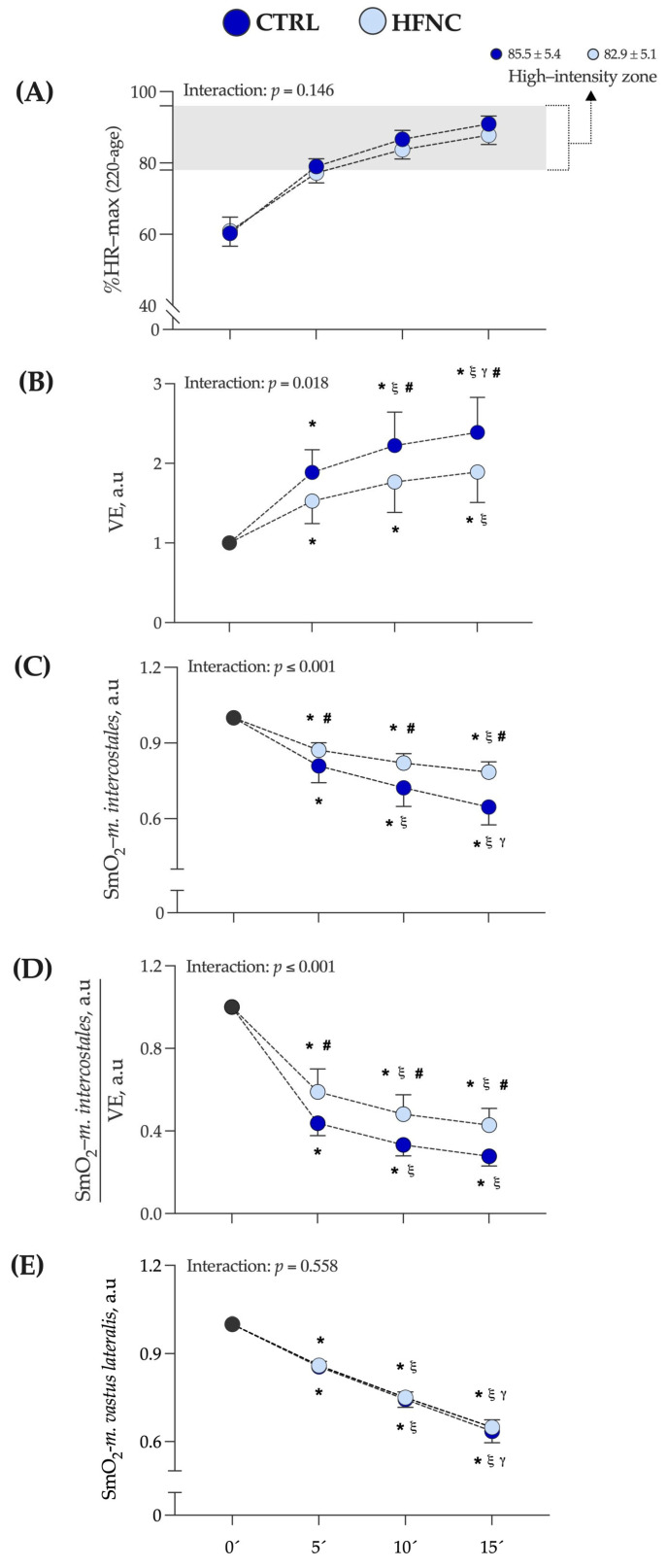
Changes in variables assessed during fifteen minutes of constant-load exercise testing at high-intensity cycling (70% of maximal power output (W max)) in conditions (CTRL vs HFNC). (**A**) %HR max: percentage of theoretical maximal heart rate according to formula 220 − age. (**B**) $\dot{V}$E: lung ventilation. (**C**) SmO_2_-*m.intercostales*: oxygen saturation levels in accessory respiratory muscles. (**D**) ratio SmO_2_-*m.intercostales*/$\dot{V}$E. (**E**) SmO_2_-*m.vastus lateralis*: oxygen saturation levels in locomotor muscles. Data are expressed as arbitrary units (a.u.) and are shown as mean and standard deviation. Changes were evaluated by two-way mixed ANOVA test (* *p* < 0.05 vs. 0′. ξ *p* < 0.05 vs. 5′. γ *p* < 0.05 vs. 10′. # *p* < 0.05 comparison CTRL vs. HFNC).

Figure 3 shows the exercise intensity at which CLETs were completed, expressed as a percentage of the maximal theoretical value of heart rate (according to formula 220 − age) (%HR max). In this study, the high-intensity exercise zone corresponds to the mean value at which the respiratory compensation point (RCP) was determined by participants in the $\dot{V}$O_2_-peak test (88.0 ± 8.0 of %HR max) [47]. The mean value at which each exercise protocol was completed corresponds to the mean of %HR max at 5, 10, and 15 min of exercise protocol in each condition (CTRL = 85.5 ± 5.4% and HFNC = 82.9 ± 5.1%).

**Figure 4:**
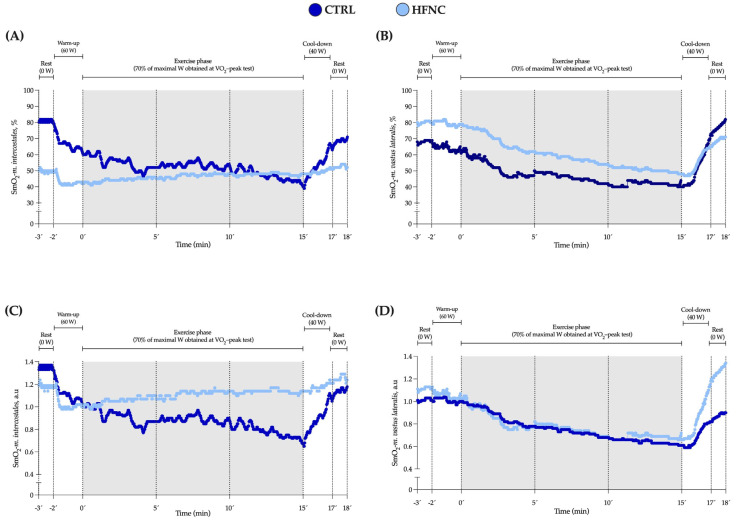
Data for SmO_2_ in respiratory (SmO_2_-*m.intercostales*) and locomotor (SmO_2_-*m.vastus lateralis*) muscles during constant-load exercise test (CLET) protocols (CTRL and HFNC) obtained from a representative participant. (**A**,**B**) raw values expressed as percent (%). (**C**,**D**) standardized values as arbitrary units (a.u.). This participant showed the highest coefficient of variation in SmO_2_-*m.intercostales* at rest (28%) but was who most significantly evidenced the effect of HFNC on SmO_2_.

We did not find a significant interaction between factors (*p* = 0.146). Also, changes in $\dot{V}$E and SmO_2_-*m.intercostales* during CLETs are shown. We observed in HFNC lower muscle desaturation in RMs levels and hyperventilation than in CTRL. There was a lower ratio between muscle oxygen saturation levels and hyperventilation when participants exercised with HFNC. During CLETs, SmO_2_-*m.intercostales* showed different changes regarding whether the exercise was completed with or without HFNC support. For SmO_2_-*m.vastus lateralis*, we did not report differences between conditions (interaction between factors: *p* = 0.558), with similar changes during exercise. Figure 4 shows an example of the changes in SmO_2_-*m.intercostales* and SmO_2_-*m.vastus lateralis* at 0′, 5′, 10′ and 15′. 

### 3.3. Changes in Symptoms and Effort Level during Exercise

**Figure 5:**
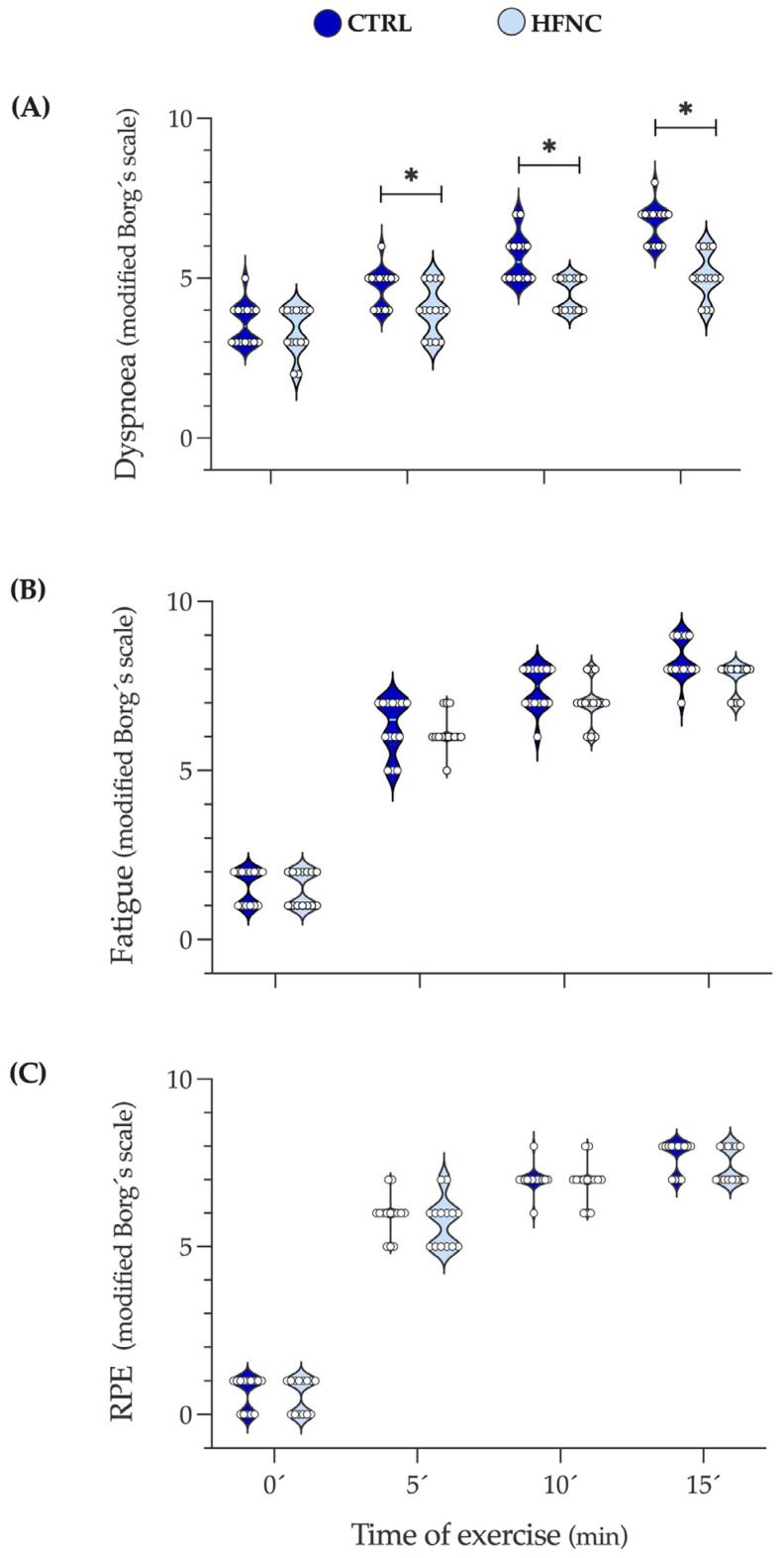
Changes in dyspnea, leg fatigue, and the level of physical effort during fifteen minutes of constant-load exercise testing at high-intensity cycling (70% of maximal power output (W max)). (**A**) Dyspnea. (**B**) Leg fatigue. (**C**) RPE: Rate of perceived exertional (level of physical effort). Data are expressed as median and interquartile range. Comparison between CLETs was evaluated by Wilcoxon matched-paired signed rank test at 0, 5, 10, and 15 min of exercise protocol (* *p* < 0.05).

Figure 5 shows the changes in dyspnea, leg fatigue, and effort levels (RPE) during exercise protocols. We observed that in HFNC, participants reported less shortness of breath than in CTRL since 5′, which can be attributable to the high airflow given by HFNC. Leg fatigue and RPE did not show differences, indicating that exercise intensity involved similar peripheral metabolic demands in both conditions. These results support the local effect of HFNC on RMs reflected in lower desaturation during exercise. 

## 4. Discussion

The study aimed to register the changes in respiratory and locomotor muscle oxygen saturation levels (SmO_2_) using optical wearable devices with NIRS technology during a high-intensity exercise protocol while non-active subjects breathed spontaneously or with high airflow administrated by HFNC. The measurements obtained help us infer the effect of workload on the cost of breathing (COB) and peripheral fatigue using an easy tool and non-invasive register method, which promotes applicability in clinical settings.

We show that in *m.intercostales* and *m.vastus lateralis*, the oxygen desaturation induced by exercise can be adequately measured by optical wearable NIRS biosensors. We also show that after cycling for fifteen minutes at intensities where the respiratory compensation point was identified, an exercise intensity associated with an elevated COB by an increase of ventilatory demand, and supported non-invasively by a high airflow, the SmO_2_ levels in *m.intercostales* are higher than without ventilatory support, with no changes in *m.vastus lateralis* desaturation levels; the lower RMs desaturation induced by exercise impact on the diminished feeling of shortness of breath. 

### 4.1. Wearable NIRS Devices and Oxygen Muscle Levels during Exercise

Previous investigations using NIRS devices have studied the changes in optical density of hemoglobin bound (or not) to oxygen in superficial muscles during exercise [32,33], expanding their utility to prescribe exercise intensity when evaluating locomotor muscles and register the progressive and continuous decline in SmO_2_ levels as the exercise intensity increases [48,49]. Also, adequate reliability in *m.intercostales* and *m.vastus lateralis* during rest and exercise have been reported previously [22,50]. 

In our study, similar results were obtained in rest conditions, with no outliers; However, in one participant, we found a coefficient of variation of 28% in SmO_2_-*m.intercostales* at rest. A possible execution of moderate-to-vigorous physical activity during the 24 h before the measurements, a different schedule on which protocols were completed, and an increase in basal metabolism could perhaps be factors that influenced the variability, which we could have identified by a continuous register of exhaled gases during the protocol (*breath-by-breath* method), completing a calorimetry assessment prior to the protocol, or measuring the level of lactate during the exercise protocol.

Our results are consistent with those previously reported by Fang et al. [20], who demonstrated that HFNC has no effect on oxygenated hemoglobin (O_2_Hb) or tissue oxygenation levels (TSI) in participants with chronic obstructive disease (COPD). However, their study showed a 2.5 µm reduction in deoxygenated hemoglobin (HHb) in the vastus lateralis muscles, which has been considered to reflect microvascular O_2_ extraction. Therefore, a reduction in HHb without a change in TSI levels could suggest that HFNC improves muscle blood perfusion. However, these results cannot be extrapolated to our study because the MOXY monitor cannot record HHb and O_2_Hb levels. We suggest that future studies incorporate other NIRS devices that can record these variables. 

In our experimental model, we did not observe differences in SmO_2_-*m.vastus lateralis* between conditions, nor in the effect of HFNC on the overload of cardiovascular variables, such as the increase in heart rate during the protocol.

In RMs, few studies have evaluated the impact of therapeutic intervention on SmO_2_ with NIRS wearable sensors [23,38,39,51]. The reason mainly given is the difficulty in recording the raw signal associated with metabolic and hematologic local changes of accessory RMs recruited while the thoracic cage is moving. Concerning this issue, devices used in this study adequately recorded changes in total hemoglobin and SmO_2_ at high exercise intensity, where both locomotor and respiratory muscles were highly demanded, particularly *m.intercostales* because of sustained high values of $\dot{V}$E during protocol, even when participants were supported by HFNC. This feature of MOXY confers its applicability in clinical settings where environmental conditions are uncontrolled or the impact of therapies on oxygen muscle levels should be known in real time (e.g., external ventilatory support, respiratory training devices, or additional supplemental oxygen support, among others). Thus, promoting the knowledge of wearable optical biosensors provided with NIRS technology to health professionals will help to expand their use and explore new scenarios.

An interesting aspect is that NIRS-based devices do not use the plethysmography principle and, thus, do not differentiate between arterial and venous blood content [52]. It gives the advantage of measuring the balance between the regional oxygen supply and demand when the cardiac pulse is abolished. However, it also has a limitation in the possibility of erroneously measuring the oxygenation state of tissues unrelated to the target [53]. This limitation measurement is sustained in the variability of shapes and dimensions of arteries, veins, capillaries, and non-vascular tissue penetrated [54]. The literature reports that baseline values vary between subjects by approximately 10% [55]. These can be seen in Figure 4A,B, where for a representative subject, the raw values of SmO_2_ are different during rest and warm-up between conditions. Thus, it is more appropriate to use tissue oximetry as a trend monitor rather than as an absolute index of tissue oxygenation.

### 4.2. Effect of HFNC on Physiological Variables, Physical Performance, and Symptoms

To our knowledge, this is the first study to report that exercise-increased COB, understood as deoxygenation of respiratory muscles and measured by optical wearable biosensors, can be decreased by the airflow supported by HFNC. The delivery’s impact of a high airflow (HF) on exercise capacity has been previously studied in clinical contexts with no conclusive results. Chatila et al. [56] compared the delivery of HF (20 L·min^−1^) vs. LF (2.5 to 6.0 L·min^−1^) airflow in patients with a diagnosis of moderate-to-severe COPD, reporting that with HF support patients were able to exercise longer and with less dyspnea. Harada et al. [14] reported similar results in patients with idiopathic pulmonary fibrosis when comparing HFNC vs. traditional oxygen therapy, but with no reduced exertional dyspnea, which are different findings to those reported by Spoletini et al. [57] in patients with cystic fibrosis about the positive effect of HFNC in exercise-induced dyspnea. 

Conversely, Suzuki et al. [58] did not find improvement in exercise capacity when patients with fibrotic interstitial lung disease completed a constant-load exercise (80% of load max achieved at CPET) with HFNC vs. Venturi mask. Similar results were reported by Vitacca et al. [19] in patients with COPD and by Chihara et al. [15] in patients with chronic respiratory failure receiving long-term oxygen therapy, even when HFNC was used during physical training sessions. 

In different clinical settings, Hui et al. [18] found that HFNC decreased the exertional dyspnea and increased exercise duration in patients with cancer but only when HF supported was complemented with oxygen (fiO_2_ > 50%). 

While most of the studies analyzed report positive effects of HF on exercise capacity, these cannot be compared to our results due to differences in the pathophysiological characteristics of the participants, the HF delivered (20 vs. 50 L·min^−1^), and the intensity of the exercise protocol (%HR max: ≈50 vs. ≈88%). Moreover, these results do not allow us to recommend using HFNC in traditional respiratory rehabilitation programs since the exercise intensities at which its usefulness was evaluated are low and very different from those usually used (between 50% and 70%). 

Our study found that the airflow provided by HFNC reduced lung ventilation ($\dot{V}$E) sufficiently to meet the respiratory demands during exercise at 5, 10, and 15 min, resulting in a lower COB than CLTR (see Figure 3B). These results complement those presented in previous studies that have shown an increase in $\dot{V}$E, which, at the cost of high recruitment of respiratory muscles (RMs), is correlated with a high COB, reflected in a reduction of ΔSmO_2_-*m.intercostales* in healthy participants [22,34]. Because of the association between ΔSmO_2_-*m.intercostales* and $\dot{VE}$, we have proposed the ΔSmO_2_-*m.intercostales*·$\dot{V}$E^−1^ ratio. It has the potential to be used in participants with chronic respiratory diseases because COB could increase prematurely during submaximal exercise, even with a low-to-moderate $\dot{V}$E, due to dysfunctions of RMs (i.e., decreased maximal inspiratory pressure and endurance) and locomotor muscles (lower type I muscle fibers and elevated H^+^ production during physical activity).

For evaluating the effect of HFNC on the main variables, we decided to compare minutes 5, 10, and 15 with the beginning of the exercise phase (0) since we expected that during the warm-up, the participants would have adapted to the high airflow provided by the HFNC, ensuring completion of the entire exercise protocol. Additionally, we chose not to compare with rest or the start of warm-up, as we did not want to evaluate when the inspiratory flow of each participant was exaggeratedly supplied by the airflow provided by the HFNC (50 L·min^−1^), thus avoiding overestimating the positive impact of HFNC on the variables evaluated during exercise.

The airflow supported by HFNC during our exercise protocol was the same for all participants, an aspect that could impact more favorably on those with higher hyperventilation induced by exercise; however, the secondary analysis did not find statistical differences. An individualized setting of HFNC considering the maximal voluntary ventilation, breathing reserve, and ventilatory changes standardized by the body surface area could minimize this methodological bias. Also, no adverse effects associated with HFNC were reported. In this regard, the use of HFNC during exercise is generally considered safe, with few reported adverse effects. Previous studies have indicated that nasal pain is the most reported adverse effect; however, this was observed in a limited number of participants and resolved promptly. Consequently, Hui et al. [59] reported good tolerability when comparing HFNC to a nasal cannula at a flow rate of 2 L·min^−1^, with no symptoms such as dry eyes, nasal dryness, or eye irritation reported.

All participants completed both CLETs, each one with fifteen minutes of constant-load exercise at high intensity to ensure a relevant increase in COB and peripheral workload, so we cannot report better exercise capacity when exercise considered the support of HFNC; however, the lowest muscular desaturation in RMs, dyspnea, and hyperventilation induced by exercise are findings that support the idea that HFNC decreases the COB associated with exercise.

### 4.3. Limitations and Directions for Future Works

This research has some limitations that should be addressed in future works. We evaluated the changes in SmO_2_ in RMs and locomotor muscles during an exercise protocol by using the MOXY^®^ device, an optical wearable biosensor with great use in sports science due to its low-cost and easy-to-use characteristics; however, it does not differentiate the changes between oxyhemoglobin ([O_2_HB]) and deoxyhemoglobin ([HHB]), variables that represent the balance between the delivery and extraction of oxygen at a muscular level [60]. In future studies, we suggest using other optical wearable biosensors that can measure hemodynamics variables to identify a possible $\dot{Q}$ delivery limitation, seen as a decrease in [O_2_HB], [HHB], and the sum of both [totalHB]. Recognizing these changes will help us to allow the changes in muscular desaturation levels to result from increased oxygen extraction by muscles and not by other changes, thereby expanding the results obtained. Likewise, another variable to consider is the penetration of the light signal to the target muscle, which is deemed half the distance between the diodes (1.5 cm for the MOXY^®^ device) [45]. We did not evaluate the adipose tissue thickness of the participants to verify the NIRS data obtained. Regardless, our participants showed normal BMI, so we consider a reduced error in recording the optical biosensors. 

Although the experimental design of this study did not consider the objective measurement of the COB (esophageal balloon catheter), and in virtue of the *good* results of our previous studies concerning the use of NIRS in RMs, we consider it appropriate to include the record of other RMs groups, such as *m.parasternal* or *m.sternocleidomastoid*, to complement the data from *m.intercostales*, especially when there is elevated hyperventilation and NIRS data on these muscles change pronouncedly [61]. 

Our participants were non-physically active but healthy subjects, so the results are especially directed to participants with similar conditions. It would have been interesting to evaluate a group of patients with CCDs to extrapolate our findings in clinical settings. Although the optical wearable biosensors used in this study showed *good* data registers in subjects with obesity, edema, sarcopenia, and diabetes, technical limitations must be considered before applying NIRS technology to assess muscular changes during therapeutic exercise interventions. 

## 5. Conclusions

During a cycling protocol of high intensity and constant load in non-active subjects, optical wearable devices based on NIRS technology allow for registering the effect of HFNC in decreasing the muscle desaturation induced by exercise in accessory respiratory muscles with no differences with or without HFNC support in locomotor muscles. This change was associated with less dyspnea without decreasing the subjective perception of lower limb fatigue and physical exertion, findings that could be attributable to the lower exercise-induced hyperventilation supported by HFNC. These results reinforce the role of HFNC as an interesting therapeutic tool in physical rehabilitation programs and expand the utility of NIRS wearable devices to monitor COB during exercise easily.

## Figures and Tables

**Table 1:** Participants characteristics (n = 18).

Variables	Mean ± Standard Deviation
Sex (n)	female = 9; male = 9
Age (years)	22 ± 2
Height (cm)	173.0 ± 5.8
Weight (kg)	65.1 ± 11.2
BMI	21.6 ± 2.8
FEV_1_ (L)	4.32 ± 0.56
FVC (L)	5.13 ± 0.78
FEV_1_ · FVC^−1^ (%)	84.2 ± 3.4
Load max (watts)	226 ± 62
$\dot{V}$O_2_-peak (mL·kg^−1^·min^−1^)	48.3 ± 6.5

Abbreviations: BMI = body mass index; FEV_1_ = Forced expiratory volume at the first second; FVC = Forced vital capacity; Load max = maximal load achieved during peak oxygen-consumption test; $\dot{V}$O_2_-peak = peak oxygen-consumption test.

## Data Availability

No new data were created or analyzed in this study. Data sharing is not applicable to this article.
